# Predicting the Match Outcome in the 2023 FIFA Women’s World Cup and Analysis of Influential Features

**DOI:** 10.5114/jhk/195563

**Published:** 2025-05-29

**Authors:** José M. Oliva-Lozano, Miguel Vidal, Farzad Yousefian, Rick Cost, Tim J. Gabbett

**Affiliations:** 1 United States Soccer Federation, Chicago, IL, United States.; 2 CIDESD, Research Center in Sports Sciences, Health Sciences and Human Development, Department of Sport Sciences, University of Beira Interior, Covilhã, Portugal.; 3 Gabbett Performance Solutions, Brisbane, QLD, Australia.

**Keywords:** monitoring, international, football, EPTS, tracking

## Abstract

The aim of this study was to build an XGBoost model to predict the match outcome and analyze match-related technical, tactical and physical performance features that may influence the predicted outcome of the match. This is an observational study which follows a retrospective design. The FIFA post-match summary reports were downloaded at the end of the 2023 Women’s World Cup and used to create a dataset which consisted of match-related technical, tactical and physical performance variables. Then, an XGBoost model was built to predict the match outcome and investigate which performance features might influence the predicted outcome of the match. The overall model achieved accuracy of 0.58 ± 0.05. Losses and wins had similar predictive accuracy (0.67 ± 0.06 and 0.67 ± 0.08, respectively), but the prediction of draws performed was significantly worse with accuracy of 0.32 ± 0.16. The top ten features for predicting wins were: (1) out to in actions by the opponent, (2) attempts at the goal, (3) in-behind actions, (4) interceptions by the opponent, (5) loose ball receptions, (6) sprinting per minute by the opponent, (7) offers received by the opponent, (8) in-front opponent, (9) interceptions, and (10) total distance per minute. The top ten features for predicting losses were: (1) attempts at the goal by the opponent, (2) interceptions, (3) out to in actions, (4) possessions interrupted, (5) loose ball receptions by the opponent, (6) in front movements, (7) distance covered by the opponent, (8) in-behind actions by the opponent, (9) total distance, and (10) sprinting per minute. In conclusion, using an XGBoost model, this is the first study to successfully predict the match outcome for wins and losses from the FIFA Women’s World Cup, but also explain which features significantly influence the prediction. This study may serve as a guide for practitioners regarding the use and application of XGBoost models in high performance.

## Introduction

In recent years, there has been a rapid development of electronic performance and tracking systems (EPTSs), including global positioning systems, local positioning systems, and optical tracking systems (Pino-Ortega et al., 2021). These EPTS have revolutionized the understanding of players' performance, with most professional soccer teams now using them on a daily basis (Paul et al., 2015; Pino-Ortega et al., 2021). Moreover, several leagues have invested in EPTSs for match-day use, benefiting both performance analysis and media purposes (Oliva-Lozano et al., 2022). Multi-camera tracking systems are examples of non-invasive EPTSs that collect technical-tactical activity and match running performance data, providing practitioners with vast amounts of data for analyzing individual and team performance, as well as performance of opposing teams (Buchheit et al., 2014). For example, FIFA post-match summary reports (FIFA, 2023), which were publicly available after each match of the 2019 Women’s World Cup, include a summary of technical/tactical and physical data for each player that participated in the match. For instance, FIFA’s Enhanced Football Intelligence metrics were provided in these reports like the phase-of-play metrics that captured teams’ tactical patterns during matches. Also, these reports inform about main physical metrics that provide insights into the total distance, which represented the cumulative distance covered across all speed zones, as well as high-intensity and sprint actions (Bradley, 2024).

Given the large amount of performance data provided by these technologies, understanding the relationship between playing performance and the match outcome is of great interest within the sports industry. However, the match outcome in soccer is a multifaceted construct which may be influenced by various factors such as shooting accuracy, specific defensive actions, set pieces, and running performance (Andrzejewski et al., 2022; Brito Souza et al., 2019; Oliva-Lozano et al., 2022). A previous study which used 2019 FIFA Women’s World Cup data indicated that winning teams generally outperformed losing teams across variables such as total passes, passing accuracy, ball possession, shots, shots on the target, ball recovery patterns, aerial challenges, and set piece indicators (Kubayi and Larkin, 2020). Additionally, Kubayi and Larkin (2020) showed that losing teams tended to lose possession more frequently, register more tackles, and receive more yellow cards compared to winning teams. Also, other studies in different male professional soccer leagues have found that variables related to shooting accuracy and ball possession are also significantly correlated with team success in professional soccer leagues (Konefał et al., 2019; Lepschy et al., 2020; Yang et al., 2018).

Furthermore, match running performance, including distance covered and efforts performed at high intensity, may significantly contribute to team success (Andrzejewski et al., 2022; Brito Souza et al., 2020; Hoppe et al., 2015; Oliva-Lozano et al., 2022; Yang et al., 2018). In the context of a regular league season, higher-ranked teams have been shown to cover shorter distances without ball possession compared to lower-ranked teams (Andrzejewski et al., 2022; Yang et al., 2018), and distance covered with ball possession, especially at high speeds, was positively associated with the total number of points obtained at the end of the season (Andrzejewski et al., 2022; Brito Souza et al., 2020). This type of research should consider a combination of match running and technical-tactical performance; however, studies using this approach are limited (Oliva-Lozano et al., 2022). From a practical standpoint, it is crucial to analyze which performance indicators should be considered not only for understanding the match outcome or success, but also for training and player development purposes (Oliva-Lozano et al., 2022).

Although there has been growing interest in performance data and outcome prediction in women’s soccer, much remains to be explored. A recent study examined competitive imbalances in the group stages of the FIFA Women’s World Cup from 1991 to 2019 (Lapré and Palazzolo, 2022). By using least squares ratings and logistic regression, the research showed how these imbalances affected teams’ chances of advancing to the knockout rounds. The findings revealed that a decrease of one goal in the group opponents’ rating could increase a team’s chances of reaching the quarterfinals by 33% (Lapré and Palazzolo, 2022). While the study did not focus on the direct outcome prediction, it highlighted how group composition imbalances significantly impacted tournament success. Moreover, a previous study analyzed position-specific distributions of player movement metrics (e.g., speed, acceleration, and tortuosity) across different phases of play and in-game win probabilities using player tracking data from all matches of the 2019 FIFA Women’s World Cup (Gregory et al., 2022). Significant differences were found in movement profiles between in-possession and out-of-possession phases, although overall positional trends across in-game win probabilities were minimal (Gregory et al., 2022).

With the abundance of data produced in modern sports events, there is growing interest in predicting outcomes and extracting valuable information, not only among sports professionals, but also the wider audience, particularly in areas such as team management and sports betting (Horvat and Job, 2020). Machine learning algorithms such as XGBoost modeling which uses a tree-based supervised machine learning technique developed from a scalable end-to-end gradient tree boosting principle have shown promise in predicting sports outcomes and analyzing performance data (Lu et al., 2021). The success of the XGBoost may be due to its scalability and ability to handle a variety of data science problems efficiently (Chen and Guestrin, 2016). Its robustness, efficiency, and flexibility allow it to outperform many other machine learning techniques, making it a good choice in different scenarios (Chen and Guestrin, 2016; Gourh et al., 2020; Lu et al., 2022). For instance, a recent study observed that when compared to other machine learning algorithms, the XGBoost achieved the best performance when predicting lower extremity muscle strain (Lu et al., 2022). Therefore, the aim of this study was to build an XGBoost model to predict the match outcome and analyze match-related technical, tactical and physical performance features that might influence the predicted outcome of the match.

## Methods

### Experimental Approach to the Problem

This study employed an observational retrospective design. The FIFA post-match summary reports were downloaded at the end of the 2023 Women’s World Cup and used to create a dataset which consisted of match-related technical, tactical and physical performance variables. Each team was categorized as a win, draw or loss according to each match outcome. Then, an XGBoost model was built to predict the match outcome and investigate which match-related technical, tactical and physical performance features might influence the predicted outcome of the match.

### Procedures

All 64 matches during the 2023 FIFA Women’s World Cup, held in Australia and New Zealand, were included in this study. The data were collected from the FIFA post-match summary reports (FIFA, 2023b), which included a summary of technical/tactical and physical data for each player that participated in the Women’s World Cup. A list of all variables, which were extracted from the FIFA post-match summary reports, and their definitions are presented in Tables 1 and 2. Additional information may be found in the FIFA’s Enhanced Football Intelligence explanation document (FIFA, 2023a). This resulted in 1898 individual observations. The data were grouped by team and match, then added to create a team summary. Opponent data for each team and match were then added as the original data only described performance of individual players. This created a dataset of 128 observations with 100 features, 28 draws, 50 losses, and 50 wins.

**Table 1A:** Definition of technical-tactical variables.

Team Feature	Opponent Feature	Definition
Passes Attempted	Passes Attempted by the Opponent	An attempted distribution action performed by a player to keep possession of the ball. A player can maneuver the ball on the ground/aerially between themselves and a team-mate.
Passes Completed	Passes Completed by the Opponent	A completed distribution action performed by a player to keep possession of the ball. A player can maneuver the ball on the ground/aerially between themselves and a team-mate.
Switches of Play	Switches of Play by the Opponent	A player attempts to pass the ball to a team-mate who is on the other side of the pitch. The ball must progress through at least two vertical field zones.
Crosses Attempted	Crosses Attempted by the Opponent	An attempted distribution action performed by a player with the intention of creating a goal scoring opportunity. The player can play the ball on the ground or aerially from any crossing zone with the intention of finding a team-mate inside the recognized target area.
Crosses Completed	Crosses Completed by the Opponent	A completed distribution action performed by a player with the intention of creating a goal scoring opportunity. The player can play the ball on the ground or aerially from any crossing zone with the intention of finding a team-mate inside the recognized target area.
Line Breaks Attempted	Line Breaks Attempted by the Opponent	Player attempts to progress the ball and break one or more unit lines of the opposition team shape.
Line Breaks Completed	Line Breaks Completed by the Opponent	A player progresses the ball and breaks one or more unit lines of the opposition team shape.
Ball Progressions	Ball Progressions by the Opponent	A distribution action by a player aiming to breach the opposition team shape by intentionally bypassing one or more players while carrying the ball into space or directly beyond an opponent.
Take Ons	Take Ons by the Opponent	A player in possession of the ball engages one or two direct opponents and attempts to directly bypass them with the ball to create retention or progression opportunities.
Step Ins	Step Ins by the Opponent	An attempt by a player to breach the opposition team shape by intentionally bypassing one or more opposition players whilst carrying the ball.
Attempts at Goal	Attempts at the Goal by the Opponent	A distribution action performed by a player with the intention of scoring a goal.
Total Offers	Total Offers by the Opponent	Number of times a player performs a deliberate action influencing the in-possession phase to receive the ball.
In Front	In Front by the Opponent	A player has moved to receive the ball in front of the opposition’s first unit and must start from beyond the oppositions first unit or the highest attacking player.
In Between	In Between by the Opponent	Player moved to receive the ball within or between two units, inside the opposition’s team shape
Out to In	Out to In by the Opponent	A player has performed a movement from outside the opposition team shape to inside the opposition team shape to receive the ball.
In to Out	In to Out by the Opponent	A player has performed a movement from inside the opposition team shape to outside the opposition team shape to receive the ball.
In Behind	In Behind by the Opponent	A player has performed a movement to receive the ball in behind the opposition final unit.
No Movement	No Movement by the Opponent	A player has provided no movement to receive the ball.
Offers Received	Offers Received by the Opponent	Number of times a player has received the ball.

**Table 1B:** Definition of technical-tactical variables.

Team Feature	Opponent Feature	Definition
Tackles Made	Tackles Made by the Opponent	An attempt by a player to dispossess their opponent.
Tackles Won	Tackles Won by the Opponent	A successful attempt by a player to dispossess their opponent.
Blocks	Blocks by the Opponent	A player attempts to stop the opposition’s in-possession action reaching its intended target without the aim of retaining possession of the ball for themselves.
Interceptions	Interceptions by the Opponent	A player anticipates where the ball is being distributed with the intention of winning possession of the ball for themselves or their team.
Pressing Direct	Pressing Direct by the Opponent	A player has directly and aggressively closed down space between themselves and the opposition player with the ball and can compete for possession.
Pressing Indirect	Pressing Indirect by the Opponent	A player may be trying to close down space and control the direction in which the opposition player in possession can move with the ball, without directly trying to regain possession.
Aerial Duels Won	Aerial Duels Won by the Opponent	The player competing in an aerial duel wins the contest for the ball.
Physical Duels Won	Physical Duels Won by the Opponent	The player competing in a physical duel wins the contest for the ball.
Possession Contests Won	Possession Contests Won by the Opponent	With the final touch before the player's possession contest event coming from the opposition team, the next touch of the ball is made by a player of its team after that contest.
Clearances	Clearances by the Opponent	A player attempts to clear the ball upfield or out of play, usually to relieve the pressure or danger.
Loose Ball Receptions	Loose Ball Receptions by the Opponent	A player takes control of the ball after neither team has had controlled possession of it.
Pushing On	Pushing On by the Opponent	A player tries to close space between themselves and an opponent when the opponent does not have the ball.
Pushing On into Pressing	Pushing On into Pressing by the Opponent	A player closed space between themselves and an opponent who does not have the ball and continued to do so after the opponent gained possession, to reduce time and space for the opponent.
Possession Regains	Possession Regains by the Opponent	A player has helped their team receive possession of the ball after a defensive event.
Possession Interrupted	Possession Interrupted by the Opponent	For the attacker performing an in-possession event (pass, cross or attempt at the goal), the ball possession has achieved its intended purpose, but with external influence. A successful turnover in possession was not achieved by the defender despite touching the ball.

**Table 2:** Definition of physical performance variables.

Team Feature	Opponent Feature	Definition
TD	TD Opponent	Total distance (m)
Z1	Z1 Opponent	Distance between 0–7 km/h (m)
Z2	Z2 Opponent	Distance between 7–13 km/h (m)
Z3	Z3 Opponent	Distance between 13–19 km/h (m)
Z4	Z4 Opponent	Distance between 19–23 km/h (m)
Z5	Z5 Opponent	Distance above 23 km/h (m).
HSR count	HSR count Opponent	Count of high-intensity runs (Z4).
SPR count	SPR count Opponent	Count of sprinting runs (Z4).
TD Per Min	TD Per Min Opponent	Total distance divided by the duration in minutes.
Z1 Per Min	Z1 Per Min Opponent	Z1 distance divided by the duration in minutes.
Z2 Per Min	Z2 Per Min Opponent	Z2 distance divided by the duration in minutes.
Z3 Per Min	Z3 Per Min Opponent	Z3 distance divided by the duration in minutes.
Z4 Per Min	Z4 Per Min Opponent	Z4 distance divided by the duration in minutes.
Z5 Per Min	Z5 Per Min Opponent	Z5 distance divided by the duration in minutes.
HSR Per Min	HSR Per Min Opponent	Count of high-intensity runs divided by the duration in minutes.
SPR Per Min	SPR Per Min Opponent	Count of sprinting runs divided by the duration in minutes.

### Data Analysis

The data were then split based on the phase of the tournament: one dataset for the group stage was used for training, while another dataset for the knockout stage was used for testing. Within the training set the results were as follows: 20 draws, 38 losses, and 38 wins. To address the impact of class imbalance and enhance the machine learning model's effectiveness, the upSample function from the caret R package was used to randomly duplicate draws to equal the number of losses and wins.

The XGBoost, which is a boosting algorithm based on ensemble learning, was selected as the chosen model for match outcome prediction based on its ability to identify relationships in high-dimensional data, handle imbalanced data, and higher accuracy compared to other algorithms (Mandorino et al., 2023). Prior to developing the XGBoost model, Recursive Feature Elimination (RFE) was used to determine the optimal subset of features. The RFE process involved 12-fold cross-validation, which was repeated three times on feature subset sizes of 50 and 70, with each model being assessed by the Kappa statistic for performance. The subset sizes of 50, 70, and 100 were chosen due to the tradeoff of computational cost and inclusion of a sufficient number of features to capture the underlying patterns in the data effectively. Consequently, it was determined that 70 of the 100 features provided better results compared to a model built with all features. After this, the grid search was used with cross validation to tune the hyperparameters of the model.

Once the model was established using RFE and hyperparameter tuning, the model was then repeated 10,000 times to assess the stability and reliability of its output, which was of concern due to the small dataset, similar to the approach by Rossi et al. (2018). With each iteration of the model, accuracy, precision, recall, F1 score, and multiclass AUC (based on the work by Hand and Till (2001))were collected. After establishing the performance of the model’s ability to predict the result of the knockout stage of the Women’s World Cup, Shapley Additive exPlanations (SHAP) were used to understand how the model made predictions. SHAP values are based on game theory where features are given values based on their contribution to the prediction (Lundberg and Lee, 2017). All analyses were performed using Rstudio (Version 2023.12.0.369, Posit Software, PBC, Boston, MA, USA) with R and Python libraries.

## Results

**Figure 1:**
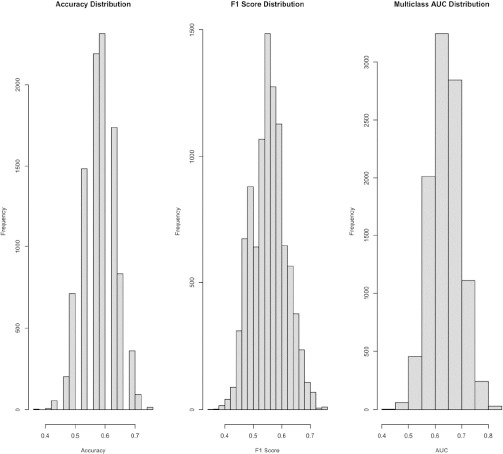
Distribution of accuracy, the F1 score, and the multiclass area under the curve (AUC) scores after 10,000 iterations of the model.

Table 3 breaks down the performance of the XGBoost model on the match outcome. The results of Table 3 summarize the mean and standard deviation of the performance metrics over 10,000 iterations, while also separating model performance of each match outcome. The model overall achieved accuracy of 0.58 ± 0.05, precision of 0.55 ± 0.7, a recall of 0.55 ± 0.06, a F1 score of 0.55 ± 0.06 and a multiclass AUC of 0.64 ± 0.06. Figure 1 shows the distribution of accuracy, the F1 score, and the multiclass AUC. This took the average of how each class performed on accuracy with losses and wins having similar accuracy, 0.67 ± 0.06 and 0.67 ± 0.08, respectively, but draws performing worse with accuracy of 0.32 ± 0.16. The model´s performance in predicting draws was consistently poor as precision (Draw = 0.35 ± 0.14, Loss = 0.64 ± 0.08, Win = 0.67 ± 0.08), recall (Draw = 0.32 ± 0.16, Loss = 0.67 ± 0.06, Win = 0.67 ± 0.08) and the F1 score (Draw = 0.34 ± 0.13, Loss = 0.65 ± 0.05, Win = 0.66 ± 0.06) were all worse when compared to predicting wins and losses.

**Table 3:** Performance of the XGBoost Model to predict the match outcome of the knockout stage of the 2023 Women’s World Cup.

	Accuracy	Precision	Recall	F1	AUC
Draw	0.32 ± 0.16	0.35 ± 0.14	0.32 ± 0.16	0.34 ± 0.13	
Loss	0.67 ± 0.06	0.64 ± 0.08	0.67 ± 0.06	0.65 ± 0.05	
Win	0.67 ± 0.08	0.67 ± 0.08	0.67 ± 0.08	0.66 ± 0.06	
Total Average	0.58 ± 0.05	0.55 ± 0.7	0.55 ± 0.06	0.55 ± 0.06	0.64 ± 0.06

*Note: AUC = area under the curve*

**Figure 2:**
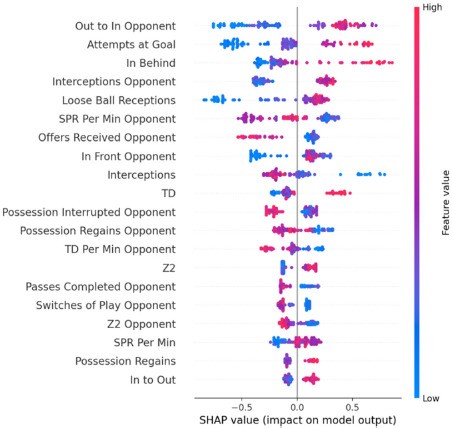
SHAP beeswarm plot of the top features that influence the model prediction for wins. The rank is based on mean SHAP value. Color represents the raw value of each feature.

Based on the consistency of the model performance with regard to wins and losses, SHAP values were calculated to determine the top features that influenced the model prediction for both match outcomes. Figure 2 shows a beeswarm plot for wins. The features are ordered top to bottom by average absolute SHAP value. Specifically, it shows that the top ten features for predicting wins were: (1) out to in by the opponent, (2) attempts at the goal, (3) in behind, (4) interceptions by the opponent, (5) loose ball receptions, (6) sprint per minute by the opponent, (7) offers received by the opponent, (8) in front opponent, (9) interceptions, and (10) total distance. The higher the SHAP value for a feature, the more influence that variable had on the model prediction for wins, thus top features were determined by the mean SHAP values where the color corresponded to the raw values of a given feature. The color of each point indicated the actual value for that feature/variable, being red/pink a high value and blue a low value. For example, looking at features such as out to in by the opponent, attempts at the goal, in behind, interceptions by the opponent, loose ball receptions, total distance, distance in zone 2, sprint per minute, possession regains and in to out, red values showed that the greater the performance in these features, the greater the influence in the model prediction for wins, while lower values might negatively influence the prediction. However, low performance/values in a specific variable might have a high SHAP value as well. For instance, sprint per minute by the opponent, offers received by the opponent, interceptions, possession interrupted by the opponent, possession regains by the opponent, total distance per minute by the opponent, passes completed by the opponent, switches of play by the opponent, or distance covered in zone 2 by the opponent positively influenced the model’s prediction of a win, while higher values in those features might negatively influence the prediction.

**Figure 3:**
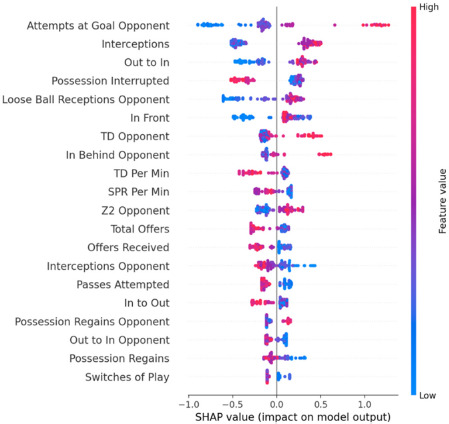
SHAP beeswarm plot of the top features that influence the model prediction for losses. The rank is based on mean SHAP value. Color represents the raw value of each feature.

Figure 3 shows a beeswarm plot for losses. The features are ordered top to bottom by average absolute SHAP value. Specifically, it shows that the top ten features for predicting losses were: (1) attempts at the goal by the opponent, (2) interceptions, (3) out to in, (4) possessions interrupted, (5) loose ball receptions by the opponent, (6) in front, (7) total distance covered by the opponent, (8) in behind by the opponent, (9) total distance per minute, and (10) sprint per minute. Looking at features such as attempts at the goal by the opponent, interceptions, out to in, loose ball receptions by the opponent, total distance covered by the opponent, in behind by the opponent, distance in zone 2 by the opponent, and possession regains by the opponent, red values showed that the greater the values in these features, the greater the influence in the model prediction for losses. However, low performance/values in a specific variable might have a high SHAP value as well. For instance, the lower the values in variables such as possession interrupted, total distance per minute, sprint per minute, total offers, offers received, interceptions by the opponent, passes attempted, in to out, out to in by the opponent, possession regains, or switches of play, the greater the influence on the prediction of losses.

## Discussion

The purpose of this study was to build an XGBoost model to predict the match outcome and analyze match-related technical, tactical and physical performance features that may influence the predicted outcome of the match. To the best of the authors’ knowledge, this is the first study building an XGBoost model, which is not only able to successfully predict the match outcome for wins and losses from the 2023 FIFA Women’s World Cup, but also explain what features influenced the prediction. One of the main findings was that for wins, the top ten features for predicting wins were: out to in by the opponent, attempts at the goal, in behind, interceptions by the opponent, loose ball receptions, sprint per minute by the opponent, offers received by the opponent, in front opponent, interceptions, and total distance. Another main finding was that, for losses, the top ten features for predicting losses were: attempts at the goal by the opponent, interceptions, out to in, possessions interrupted, loose ball receptions by the opponent, in front, total distance covered by the opponent, in behind by the opponent, total distance per minute, and sprint per minute. However, performance of the model with regard to predicting draws was consistently poor. This could be in part due to the small dataset, as well as due to the measures involved in a draw being similar to both losses and wins, meaning the model was effectively guessing as a prediction.

One of the main findings of this study was that building an XGBoost model allowed us to successfully predict the match outcome for wins and losses from the 2023 FIFA Women’s World Cup. The model achieved accuracy of ~0.58 for predicting the match outcome. The XGBoost typically employs a tree-based supervised machine learning approach, which originates from a scalable end-to-end gradient tree boosting principle (Lu et al., 2021). Typically, commonly used feature engineering methodologies encompass technical analysis and statistical methodologies (Horvat and Job, 2020). On the one hand, statistical methodologies primarily focus on compression and dimension reduction techniques (Horvat and Job, 2020). On the other hand, technical analysis operates under the assumption that future events are correlated with historical patterns (Horvat and Job, 2020). However, although a previous literature review about soccer prediction using the XGBoost algorithm explained that the current models estimated the results roughly with accuracy of about 50% (Gourh et al., 2020), these models may perform better than random guessing. In fact, they proposed a model that could perform better than many other models using the XGBoost algorithm. In this regard, a previous study highlighted the importance of incorporating a larger dataset and more variables in such analyses, as doing so often leads to enhanced model accuracy (de Jong et al., 2020).

In addition, this XGBoost model was not only able to successfully predict the match outcome for wins and losses from the 2023 FIFA Women’s World Cup, but also explain what features influenced the prediction. Our results are thought provoking considering that variables related to the offensive phase such as attempts at the goal, in-behind actions, and in to out movements were relevant features for winning prediction. Also, defensive actions such as possession regains and physical output (e.g., distance covered and sprint per minute) were determinant predictors for winning matches. Since this is the first study building an XGBoost model in order to predict the match outcome for wins and losses from the 2023 FIFA Women’s World Cup based on match technical, tactical, and physical performance variables, limited comparable data for discussion are available. A previous study, which compared technical performance of successful and unsuccessful teams (i.e., teams in the top two positions in their groups vs. bottom two positions) in the 2019 Women's World Cup, found that winning teams had greater possession, passes, accurate passes, shots, and shots on the target, and had more successful aerial challenges, ball recoveries, and corner kicks compared to losing teams (Atasever and Kiyici, 2023). In this sense, some of these key features align with our findings (e.g., importance of completed passes, attempts on the target and possession regains). Some of the features that had a high influence on the model prediction for wins were also found in previous research in men’s and women’s soccer. A previous study, which analyzed data from various professional leagues and tournaments (including Women’s World Cup data) resulting in 695 matches considered, modelled the match outcome using variables from each analytical approach, specifically using generalized linear modelling and decision trees (de Jong et al., 2020). Their findings indicate that rational and data-driven approaches surpassed the literature-driven approach in predicting match outcomes (de Jong et al., 2020). Specifically, the strongest determinants of the match outcome were scoring first, intentional assists relative to the opponent, the percentage of shots on the goal saved by the goalkeeper relative to the opponent, shots on the goal relative to the opponent and the percentage of successful duels (de Jong et al., 2020). All these variables may not match with our findings (i.e., we observed similar findings only with regard to shots on the goal), but it is also important to note that different methodological approaches were followed. Another study in a different competition (i.e., Spanish first and second division – men’s soccer) completed a factor analysis and showed that the factor which was correlated with goals scored, possession ending with a goal, shots on the target, goals from set plays, goals from a direct free kick, offsides, and goals conceded, were the most important contributors to the teams’ success (β = 0.66) (Oliva-Lozano et al., 2022). Once again, attempts at the goal is a highlighted variable not only by our study, but also by previous research (Oliva-Lozano et al., 2022). Moreover, this study found a significant interaction (*p* = 0.001) between the second division and the factor 2, which correlated with total distance covered, sprinting distance, and the number of sprints when the opposing team owned the ball, shots inside the box, tackles, and fouls received (Oliva-Lozano et al., 2022). In this sense, there is similarity in findings related to physical output variables (e.g., distance covered and sprinting actions) (Oliva-Lozano et al., 2022). Although each competition may be very context-specific when determining team success, our study has also observed that not only technical-tactical performance variables were relevant, but also physical performance (with special reference to intensity-related variables such as total meters per minute and sprinting distance per minute). These findings indicate the key role of physicality in the current female game when it comes to the team’s success. However, caution should be taken when interpreting these results considering that there is conflicting evidence regarding the impact of physical output on the match outcome (Andrzejewski et al., 2022; Chmura et al., 2022; Oliva-Lozano et al., 2022; Vescovi and Falenchuk, 2019). For example, there are several studies highlighting a potential positive impact of physical output on the team’s success (Andrzejewski et al., 2022; Chmura et al., 2022; Oliva-Lozano et al., 2022), while it has also been observed that there may be no significant differences between match outcomes in most physical performance variables (Barthelemy et al., 2024; Vescovi and Falenchuk, 2019).

This study presents some limitations which need to be acknowledged. Firstly, the sample size was confined to a tournament where the number of observations per team was limited to the outcome and qualification to subsequent rounds. This implies that the number of observations by the match outcome is different (e.g., significantly less draws than wins or losses) and there may be teams with more observations than others because of qualifying to the next round. Secondly, the quality of data captured can be improved to provide a greater context to the information captured. For example, understanding where on the field interceptions occurred would help indicate if turnovers happened closer or further away from a team’s goal. In this regard, the model can improve with greater data quantity and quality. Performance of the model with regard to predicting draws was consistently poor, which could be because of a lower sample size. Alternatively, because a drawn match is situated right in the middle of winning and losing, it may be harder to predict such match outcomes. Moreover, the practice of grouping match observations solely by the match outcome may be overly simplistic, failing to capture the dynamic nature of the evolving match status during observations. For example, a team could score in the final minutes of a match after being tied for most of the game, yet still be classified as a win. Future research should explore the impact of evolving match status (i.e., drawing, winning, or losing) on performance. Furthermore, while the XGBoost model used in this study offers high predictive accuracy concerning wins and losses, there are some drawbacks to using this model. These drawbacks include interpretability issues compared to simpler models and the potential for overfitting, especially when the model is exposed to unseen data. Overfitting is a particular concern when it comes to hyperparameter tuning.

## Practical Implications

This study may serve as a guide for practitioners regarding the use and interpretation of XGBoost models in high performance settings when dealing with a small and imbalanced dataset with several features through utilizing RFE, grid search hyperparameter tuning, and SHAP values for model interpretation. This technique is recommended for establishing the top features for wins and losses given the achieved accuracy (~0.67 for both) to predict the match outcome and ease to communicate results to sport staff. However, it is important to understand that there are limitations to the model, and it can be improved upon as mentioned above.

In addition, understanding match-play characteristics is crucial for guiding practices within women's soccer, thus this study represents an initial effort to consolidate scientific literature assessing the match demands of women's soccer and understanding the attributes of success in an international tournament of this magnitude. It is recommended that practitioners use as a reference the SHAP beeswarm plots of the top features that influence the model prediction for wins (Figure 2) and losses (Figure 3) at the FIFA 2023 Women’s World Cup. By focusing on performance metrics and understanding their impact on match outcomes, coaches can develop more effective training programs and strategic plans. For example, if variables such as attempts at the goal are key features associated with winning, emphasis on attacking drills that improve shooting accuracy and frequency may be necessary. In addition, training “in behind” actions (i.e., players performing movements to receive the ball in behind the opposition final unit), which is also associated with winning the match, may be a proxy to increase attempts at the goal. However, if attempts at the goal by the opponent are associated with losing, it may be necessary to strengthen defensive strategies to limit opponents' shooting opportunities.
